# French national epidemiology of bacterial superinfections in ventilator-associated pneumonia in patients infected with COVID-19: the COVAP study

**DOI:** 10.1186/s12941-023-00603-0

**Published:** 2023-06-28

**Authors:** Maxime Pichon, Julie Cremniter, Christophe Burucoa, the COVAP Study group, Sahar Abdallah, Corentine Alauzet, Tom Alix, Kahina Allouche, Marlène Amara, Florence Anglade, Nadia Anguel, Laurence Armand-Lefevre, Francois Barbier, Clémence Beauruelle, Pascale Bemer, Hanaa Benmansour, Béatrice Bercot, Ludovic Bergon, Dominique Bertei, Marc Berthon, Pascal Beuret, Léa Bientz, Laura Billon, Aurore Bousquet, Amélie Brousse, Lauranne Broutin, Fabrice Bruneel, Anne Cady, Francois Camelena, Amélie Carrer-Causeret, Yvan Caspar, Lotfi Chemali, Anne Christine Jaouen, Théophile Cocherie, Aurélie Cointe, Stephane Corvec, Laura Courtellemont, Gaelle Cuzon, Anne Dao, Agathe Delbove, Camille D’Humieres, Laura Djamdjian, Alexandra Doloy, Joséphine Dorin, Yann Dumont, Bruno Dumoulard, Faten El Sayed, Marie-Sarah Fangous, Laurent Favier, Alexis Ferre, Nicolas Fortineau, Juliette Francois, Clémence Gachet, Mahmoud Gargouri, Denis Garot, Nabil Gastli, Elena Gauvin, Isabelle Geneau, Guillaume Geslain, Antoine Goury, Romaric Grenot, Antoine Grillon, Thomas Guillard, Aurélie Guillouzouic, Jerome Guinard, Jennifer Guiraud, Esther Gyde, Christophe Henry, Katy Jeannot, Marie Kempf, Achille Kouatchet, Luce Landraud, Philippe Lanotte, Sebastien Larreche, Brice Le Gallou, Elodie Le Breton, Pierre-Etienne Leblanc, Hervé Lecuyer, Ludovic Lemee, Pauline Lessard, David Leyssene, Pierre Lureau, Anne-Elisabeth Manteaux, Michael Mervent, Maite Micaelo, Anthony Michaud, Olivier Moquet, Anaelle Muggeo, Evelina Ochin, Patrick Ochocki, Abdelali Ouchikhe, Maxime Paluch, Marie Pancher-Lory, Alix Pantel, Adeline Pastuszka, Ophélie Perruche, Olivia Peuchant, Caroline Piau, Chloé Plouzeau-Jayle, Kevin Quesnel, Lucie Richard, Emeline Riverain, Alexandre Robert, Anne-Laure Roux, Pierre Saint-Sardos, Laurent Serpin, Daniel Silva, Valerie Sivadon-Tardy, Karim Toumert, Céline Tournus, Pauline Touroult-Jupin, Antoine Tran Quy, Anne Vachee, Christian Vanjak, Véronique Vernet-Garnier, Camille Vinclair, Jérémie Violette, Violaine Walewski

**Affiliations:** 1 CHU Poitiers , Infectious Agents Department. Bacteriology and Infection Control Laboratory, 2 rue de la Milétrie, 86021 Poitiers, France; 2 Université de Poitiers, INSERM. U1070 Pharmacology of Antimicrobial Agents and Antibiotic Resistance, Medicine and Pharmacy University, Poitiers, France

**Keywords:** Ventilator-associated pneumoniae, Bacterial superinfection, Antibiotic resistance, Intensive care, COVID-19

## Abstract

**Background:**

Description and comparison of bacterial characteristics of ventilator-associated pneumonia (VAP) between critically ill intensive care unit (ICU) patients with COVID-19-positive, COVID + ; and non-COVID-19, COVID-.

**Methods:**

Retrospective, observational, multicenter study that focused on French patients during the first wave of the pandemic (March–April 2020).

**Results:**

935 patients with identification of at least one bacteriologically proven VAP were included (including 802 COVID +). Among Gram-positive bacteria, *S. aureus* accounted for more than two-thirds of the bacteria involved, followed by *Streptococcaceae* and *enterococci* without difference between clinical groups regarding antibiotic resistance. Among Gram-negative bacteria, *Klebsiella* spp. was the most frequently observed bacterial genus in both groups, with *K. oxytoca* overrepresented in the COVID- group (14.3% *vs*. 5.3%; p < 0.05). Cotrimoxazole-resistant bacteria were over-observed in the COVID + group (18.5% *vs*. 6.1%; p <0.05), and after stratification for *K. pneumoniae* (39.6% *vs*. 0%; p <0.05). In contrast, overrepresentation of aminoglycoside-resistant strains was observed in the COVID- group (20% *vs*. 13.9%; p < 0.01). *Pseudomonas* sp. was more frequently isolated from COVID + VAPs (23.9% *vs*. 16.7%; p <0.01) but in COVID- showed more carbapenem resistance (11.1% *vs*. 0.8%; p <0.05) and greater resistance to at least two aminoglycosides (11.8% *vs*. 1.4%; p < 0.05) and to quinolones (53.6% *vs*. 7.0%; p <0.05). These patients were more frequently infected with multidrug-resistant bacteria than COVID + (40.1% *vs*. 13.8%; p < 0.01).

**Conclusions:**

The present study demonstrated that the bacterial epidemiology and antibiotic resistance of VAP in COVID + is different from that of COVID- patients. These features call for further study to tailor antibiotic therapies in VAP patients.

**Supplementary Information:**

The online version contains supplementary material available at 10.1186/s12941-023-00603-0.

## Introduction

Ventilator-associated pneumonia (VAP) is the second most common nosocomial infection and remains the leading cause of death in critically ill patients [1]. Prior to COVID, the risk of VAP was estimated to be 1.5% per mechanical-ventilation-day, decreasing to < 0.5% daily after 2 weeks of mechanical ventilation, and was associated with a 7 day increase in hospital length of stay and a $40,000 increase in healthcare costs [2].

Since the beginning of the COVID pandemic, significant disparities in prevalence of VAP have been demonstrated. For example, a prevalence rate of 29% in Italy was reported in the first wave, while other countries reported an incidence of 79% (HR of 2.1 to COVID-negative patients) [3, 4]. In this context, it cannot be ruled out that the incidence ratio and the representation of the epidemiology of bacterial superinfections are biased.

Before COVID, the microbial etiology of VAPs varied according to duration of ventilation, length of hospital stays and the local microbial ecology (reflecting local antibiotic prescribing habits). VAPs were caused by Gram-negative bacteria (GNB, mainly *Escherichia coli, Klebsiella pneumoniae*, and *Pseudomonas aeruginosa*), accounting for 50–80% of cases, to a greater extent than Gram-positive bacteria (GPB, *Staphylococcus aureus* and *Streptococcaceae*), which accounted for < 40% of cases [5, 6].

The common problem of antimicrobial stewardship for bacterial co-/super-infection in patients with severe SARSCoV2 infection, particularly those requiring intensive care and ventilation, remains a challenge. The French Society of Anesthesia and Resuscitation recommended that these critical care and ventilation issues could be avoided by optimizing the clinical management of patients [7, 8]. The society emphasized the value of limiting the duration of ventilation and of requesting microbial specimens whenever possible in cases of clinical suspicion requiring any type of respiratory specimen for the diagnosis of microbial infections [9, 10]. Optimization of antibiotic therapy in suspected VAP, which is crucial for proper clinical management of the disease, depends on the risk factor for the carriage of multiresistant bacteria (MDR), and cannot be limited to an unwarranted combination of antibiotics [8, 11].

The objective of this study, conducted at the very beginning of the French COVID pandemic, was to compare the bacterial characteristics of VAP in critically ill patients infected with COVID-19, COVID + ; and uninfected, COVID-, admitted to intensive care units (ICU) during the same period.

## Methods

### Design

This study is a retrospective, observational, multicenter study that focused on French patients during the first French wave of the pandemic. All voluntary-based participating centers were contacted through professional networks (Collège de Bactériologie, Virologie et Hygiène Hospitalière, and Société Française de Microbiologie, SFM). The investigators retrospectively analyzed the clinical records to complete an electronic clinical record file that was blinded by the principal investigators.

### Participants

This study included all patients hospitalized in French intensive care units (private, general, military, and university hospitals) who required mechanical ventilation during the first month of the first French wave of the COVID pandemic (March to April 2020). All patients in whom the diagnosis of VAP was established bacteriologically (“identification of a bacterium at a concentration above a threshold depending on the nature of the respiratory specimen in which it was identified”) before introduction of antibiotics were included [12]. The application of these thresholds allowed to differentiate between infection (above the threshold) and colonization (below the threshold). The CDC VAP 2020 diagnostic guidelines, adapted in the present study, required at least one of the following [13]: i/Fever ≥ 38.5 ℃; ii/Leukopenia or leukocytosis (white blood cell count ≤ 4 G/L or ≥ 12G/L); and two of the following: i/Change in sputum character, or newly purulent sputum; ii/Increased airway secretions or need for aspiration; iii/New or worsening cough, tachypnea, or dyspnea; iv/Noisy breathing or abnormal bronchial noise; v/Poor gas exchange possibly requiring more oxygen or the use of a ventilator. In addition, and as mentioned in the above guidelines, it should be noted that to be considered, the patient must also have been on mechanical ventilation for at least two calendar days, and mechanical ventilation must be present either on the day of diagnosis of pneumonia or the day before.

### Microbiological diagnosis

All samples were analyzed according to SFM standards, and concentrations of the respective bacteria were interpreted according to the recommended thresholds (10^5^ CFU/mL for aspirates, 10^4^ CFU/mL for bronchoalveolar lavage, 10^3^ CFU/mL for the protected distal sample). Antibiotic susceptibility testing (AST) (disc diffusion or liquid dilution) was interpreted according to the thresholds and recommendations of the European Committee for Antimicrobial Susceptibility Testing (EUCAST).

### Objective of the study and evaluation criteria

The primary objective was to evaluate the prevalence of above-the-threshold bacteria responsible for superinfection in mechanical ventilation-requiring COVID +. The secondary objectives were to describe the AST of these bacteria before any antibiotic therapy.

According to the French Society of Hospital Hygiene, MDR included methicillin-resistant *S. aureus* (MRSA), extended-spectrum-βlactamase/Carbapenemase-producing E*nterobacterales* (E-ESBL/CPE), ceftazidime-resistant *P. aeruginosa*, carbapenem-resistant *Acinetobacter baumannii* (CRAB), and glycopeptide-resistant *Enterococcus faecium/faecalis* (GRE) [14, 15].

### Statistical methods

Descriptive statistics were expressed as percentages for categorical variables and as mean with standard deviation or median with interquartile range for continuous variables. Demographic characteristics, underlying conditions, and clinical characteristics were functions of COVID status using Pearson’s chi-square test or Fisher’s exact test (GraphPad Prism v9.0.0). Results with p < 0.05 were considered as significant.

### Ethical procedure

All samples were pseudonymized during completion of the eCRF by local investigators prior to collection and analyses by the national coordinators. This study was authorized by the Commission Nationale de l'Informatique et des Libertés (CNIL, n°920232) and informed consent was waived for this particular study (due to the context of the first wave).

## Results

### Clinical and demographic characteristics of patients at diagnosis

During the study period, 935 patients with identification of at least one bacteriologically proven VAP from 65 hospital centers (including 802 COVID + ; Table 1) were included. Regarding age distribution, COVID + were significantly older than COVID- (70.0 *vs*. 60.4 years; p < 0.01), without difference in sex (3.7 men per woman). As expected, the proportion of patients with a risk factor for severe COVID (as defined by French national authorities, Haut Conseil de la Santé Publique, March 14, 2020) was higher in COVID + than in COVID- (72.7% *vs*. 62.1%; p < 0.05), with more cardiovascular disease (34.9% *vs*. 29.7%; p < 0.05), diabetes mellitus (16.8% *vs*. 6.1%; p < 0.01) but less chronic respiratory disease (and/or severe liver disease; 0.1% *vs*. 4.7%; p < 0.01) in COVID + [16]. Of note, the number of risk factors per patient was similar between groups (1.69 *vs*. 1.80). VAP was discovered earlier in the clinical history of COVID + with a time lapse between intubation and the first positive specimen of 8.9 days (*vs*. 12.3; p < 0.05) without differential time lapse between the groups regarding the time between symptomatology and specimen, or symptomatology and intubation. The nature of the respiratory samples, taken before antibiotic therapy, differed between the groups, with a greater need for distal samples (25.8 *vs*. 14.4; p < 0.01) in COVID +, with no difference in the number of pathogenic bacteria identified. Finally, over the course of the patients’ clinical history, COVID + had longer duration of hospitalization than COVID- (31 days *vs*. 22 days; p < 0.01) with a lower rate of return home (7.9% *vs*. 13.0%; p < 0.01) but shorter duration of intubation (15 *vs*. 21 days; p < 0.01). No difference in case fatality rate could be observed in this cohort (33.8% *vs*. 38.2%).

**Table 1:** Patient characteristics at baseline and during clinical history

Patients	Overall (n = 935)	COVID + patients (n = 803)	COVID- patients (n = 132)	p-value
Age (years) (mean; IQR)	62.6 [56;71]	70.0 [56;71]	60.4 [50;71]	< 0.05
18–30 (n. %)	12 (1.3)	5 (0.6)	7 (5.3)	< 0.05
31–45 (n. %)	78 (8.3)	60 (7.5)	18 (13.6)	< 0.05
46–60 (n. %)	252 (26.8)	225 (28.0)	26 (19.7)	–
61–75 (n. %)	478 (51.1)	421 (52.4)	56 (42.4)	< 0.05
> 75 (n. %)	109 (11.6)	89 (11.1)	20 (15.2)	< 0.05
Unknown	8 (0.9)	3 (0.4)	5 (3.8)	–
Sex ratio (M/F)	738/196	635/167	103/29	–
Risk factors Y/N (n. %)	666 (71.2)	584 (72.8)	82 (62.1)	< 0.05
Risk factors (mean; IQR)	1.71 [1;2]	1.69 [1;2]	1.80 [1;2]	–
Age ≥ 70 years	246 (36.9)	214 (36.6)	32 (39.0)	–
History of cardiovascular disease	388 (58.3)	344 (58.9)	44 (53.6)	< 0.05
Diabetes mellitus or complication	175 (26.3)	166 (28.4)	9 (11.0)	< 0.05
Chronic respiratory disease	120 (18.0)	94 (16.1)	26 (31.7)	< 0.05
Terminal renal failure	20 (3.0)	19 (3.2)	1 (1.2)	–
Under treatment cancer	29 (4.3)	22 (3.8)	7 (8.5)	–
Immunosupression/depression	57 (8.6)	48 (8.2)	9 (11.0)	–
Child B or higher cirrhosis	8 (1.2)	1 (0.2)	7 (8.5)	< 0.05
BMI > 30	91 (13.7)	78 (13.4)	13 (15.9)	–
Delay (days; mean; IQR)				
COVID symptoms—sampling	20.2 [11;21]	20.9 [12;21]	16.05 [3.75; 24.5]	–
Intubation–sampling	9.4 [5;12]	8.9 [5;12]	12.33 [3.5;17]	< 0.05
COVID symptoms—intubation	10.9 [4;10]	12.0 [5;10]	3.67 [0;4]	–
Sampling nature (n. %)				< 0.05
Nasopharyngeal aspirates	16 (1.7)	14 (1.7)	2 (1.5)	–
Tracheal aspirates	468 (50.1)	396 (49.3)	72 (54.6)	–
Expectoration	40 (4.3)	30 (3.7)	10 (7.6)	–
Broncho-alveolar lavage	185 (19.8)	156 (19.4)	29 (22.0)	–
Protected distal sampling	226 (24.2)	207 (25.8)	19 (14.4)	< 0.05
Multiple bacteria (n. %) (n = 415)	414 (44.4)	351 (43.7)	63 (47.7)	–
Bacteria number per positive sample (moy. IQR)	1.0 [1;2]	1.0 [1;2]	1.0 [1; 2]	–
Length of (mean number of days; IQR)				
Intubation	15.4 [11;19]	15.0 [11;19]	17.1 [9.3; 22]	< 0.05
Hospitalization	21.8 [15;26]	20.9 [15;25]	27.9 [15;33]	< 0.05
Status at the end date (n. %)				
Death	306 (32.7)	261 (32.5)	45 (38.2)	–
ICU	79 (8.8)	69 (8.6)	10 (8.1)	–
Hospitalization	392 (44.1)	345 (43.0)	47 (38.2)	–
Rehabilitation	39 (4.4)	36 (4.5)	3 (2.4)	–
Home return	77 (8.6)	61 (7.6)	16 (13.0)	< 0.05
Unknown	42 (4.5)	31 (3.9)	11 (8.3)	–

### Bacterial epidemiology

During the study period, 950 pathogenic bacteria were considered to be involved in VAP, including 802 in COVID + (Table 2). Without difference in proportion between the two groups, GPB represented approximately one quarter of the bacteria identified (24.8% *vs*. 27.0%) while GNB represented most isolates (75.2% *vs*. 73.0%).

**Table 2:** Identified bacteria in diagnosis sample

Micro-organisms	Overall (n = 950)	COVID + patients (n = 803)	COVID- patients (n = 148)	p-value
Gram-positive bacteria (n; % of the group)	239 (25.1)	199 (24.8)	40 (27.0)	–
*Staphylococcus* sp. (n; % of the Gram-positive bacteria)	166 (69.5)	139 (69.8)	27 (67.5)	–
*S. aureus* (n; % of the Staphylococci)	155 (93.4)	130 (93.5)	25 (92.6)	–
Coagulase-negative staphylococci (CoNS; n; % of the Staphylococci)	11 (6.6)	9 (6.5)	2 (7.4)	
*S. epidermidis* (n; % of the CoNS)	8 (72.7)	6 (66.7)	2 (100)	–
*S. haemolyticus* (n; % of the CoNS)	2 (18.2)	2 (22.2)	–	
*S. lugdunensis* (n; % of the CoNS)	1 (9.1)	1 (11.1)	–	
*Streptococcus sp*. (n; % of the Gram-positive bacteria)	34 (14.2)	27 (13.6)	7 (17.5)	–
*S. pneumoniae* (n; % of the Streptococci)	15 (44.1)	11 (40.7)	4 (57.1)	–
*S. agalactiae* (n; % of the Streptococci)	3 (8.8)	2 (7.4)	1 (14.3)	
*S. pyogenes* (n; % of the Streptococci)	1 (2.9)	–	1 (14.3)	
Other (n; % of the Streptococci)	15 (44.1)	14 (51.9)	1 (14.3)	
*S. pseudopneumoniae*	1	1	–	–
*S. milleri* group	14	13	1	
*Enterococcus sp*. (n; % of the Gram-positive bacteria)	29 (12.1)	23 (11.6)	6 (15.0)	–
*E. faecalis* (n; % of the Enterococci)	26 (89.7)	22 (95.7)	4 (66.7)	< 0.05
*E. faecium* (n; % of the Enterococci)	3 (10.3)	1 (4.3)	2 (33.3)	
*Corynebacterium sp*. (n; % of the Gram-positive bacteria)	8 (3.3)	7 (3.5)	–	–
*C. accolens* (n; % of the *Corynebacteria*)	2 (25.0)	2 (28.6)	–	–
*C. amycolatum* (n; % of the *Corynebacteria*)	1 (12.5)	1 (14.3)	–	
*C. striatum* (n; % of the *Corynebacteria*)	4 (50.0)	4 (57.1)	–	
Other^a^				
Gram-negative bacteria (n; % of the group)	711 (74.8)	604 (75.2)	108 (73.0)	–
*Klebsiella* sp. (n; % of the Gram-negative bacteria)	153 (21.5)	132 (21.9)	21 (19.4)	–
*K. aerogenes* (n; % of the *Klebsiella* spp.)	73 (47.7)	66 (50.0)	7 (33.3)	< 0.05
*K. pneumoniae* complex (n; % of the *Klebsiella* spp.)	65 (42.5)	55 (41.7)	10 (47.6)	
*K. oxytoca* (n; % of the *Klebsiella* spp.)	10 (6.5)	7 (5.3)	3 (14.3)	
*K. varicola* (n; % of the *Klebsiella* spp.)	4 (2.6)	4 (3.0)	–	
*K. ornithinolytica* (n; % of the *Klebsiella* spp.)	1 (0.7)	–	1 (4.8)	
*Escherichia coli* (n; % of the Gram-negative bacteria)	84 (11.8)	70 (11.6)	14 (13.0)	–
*Enterobacter* sp. (n; % of the Gram-negative bacteria)	65 (9.1)	55 (9.1)	10 (9.3)	–
*E. cloacae complex* (n; % of the *Enterobacter* spp.)	63 (96.9)	53 (96.4)	10 (100)	
*E. bugandensis* (n; % of the *Enterobacter* spp.)	2 (3.1)	2 (3.6)	–	–
*Serratia marcescens* (n; % of the Gram-negative bacteria)	42 (5.9)	33 (5.5)	9 (8.3)	–
*Proteus* sp. (n; % of the Gram-negative bacteria)	34 (4.8)	26 (4.3)	8 (7.4)	–
*P. mirabilis* (n; % of the *Proteus* spp.)	29 (85.3)	21 (80.8)	8 (100)	–
*P. vulgaris* (n; % of the *Proteus* spp.)	3 (8.8)	3 (11.5)	–	
*P. hauseri* (n; % of the *Proteus* spp.)	1 (2.9)	1 (3.8)	–	
*P. penneri* (n; % of the *Proteus* spp.)	1 (2.9)	1 (3.8)	–	
*Citrobacter koseri* (n; % of the Gram-negative bacteria)	29 (4.1)	26 (4.3)	3 (2.8)	–
* Hafnia alvei* (n; % of the Gram-negative bacteria)	24 (3.4)	21 (3.5)	3 (2.8)	–
* Morganella morganii* (n; % of the Gram-negative bacteria)	20 (2.8)	18 (3.0)	2 (1.9)	–
* Pseudomonas* sp. (n; % of the Gram-negative bacteria)	162 (22.8)	144 (23.8)	18 (16.7)	< 0.05
*P. aeruginosa* (n; % of the *Pseudomonas* spp.)	159 (98.1)	141 (97.9)	18 (100)	–
*Pseudomonas* sp. (excl. *P. aeruginosa*) (n; % of the *Pseudomonas* spp.)	3 (1.9)	3 (2.1)	–	
*Acinetobacter* sp. (n; % of the Gram-negative bacteria)	21 (3.0)	19 (3.1)	2 (1.9)	–
*A. baumannii* (n; % of the *Acinetobacter* spp.)	14 (66.7)	12 (63.2)	2 (100)	–
*Acinetobacter* sp. (excl. *A. baumannii*) (n; % of the *Acinetobacter* spp.)	7 (33.3)	7 (37.8)	–	
*Stenotrophomonas maltophilia* (n; % of the Gram-negative bacteria)	25 (3.5)	21 (3.5)	4 (3.7)	–
*Achromobacter dentrificans/xylosoxidans* (n; % of the Gram-negative bacteria)	5 (0.7)	4 (0.7)	1 (0.9)	–
*Haemophilus* sp. (n; % of the Gram-negative bacteria)	35 (4.9)	26 (4.3)	9 (8.3)	–
*H. influenzae* (n; % of the *Haemophilus* spp.)	33 (94.3)	24 (92.3)	9 (100)	–
*Haemophilus* sp. *(excl. H. influenzae)* (n; % of the *Haemophilus* spp.)	2 (5.7)	2 (7.7)	–	
*Moraxella catarrhalis* (n; % of the Gram-negative bacteria)	3 (0.4)	2 (0.3)	1 (0.9)	–
*Burkholderia* sp. (n; % of the Gram-negative bacteria)	5 (0.7)	5 (0.8)	–	–
*B. cepacia* (n; % of the *Burkoholderia* spp.)	3 (60.0)	3 (60.0)	–	–
*B. gladioli* (n; % of the *Burkoholderia* spp.)	2 (40.0)	2 (40.0)	–	–
Other^b^(n; % of the Gram-negative bacteria)	4 (0.6)	2 (0.3)	2 (1.9)	–

^a^Gram-positive other bacteria: *Alloscardovia omnicolens*, *Lactobacillus casei*

^b^Gram-negative other bacteria: *Neisseria* sp., *Prevotella* sp., *Sphingomonas paucimobilis*

Among GPB, in both COVID + and COVID-, *staphylococci* accounted for more than two-thirds of the bacteria involved (69.9% *vs*. 67.5%), followed by *Streptococcaceae* (13.57% *vs*. 17.50%) including *pneumococci* (32.4% *vs*. 57.1% of *Streptococcaceae*) followed by *enterococci* (11.6% *vs*. 15.0% of GPB). *Enterococci* isolation was more frequently due to *Enterococcus faecium* in COVID- (3.5% *vs*. 33.3%; p < 0.05).

Among *Enterobacterales*, *Klebsiella* sp. was the most frequently observed bacterial genus in both groups (21.9% *vs*. 19.4%), with greater representation of *K. oxytoca* isolated in COVID- (14.3% *vs*. 5.3%; p < 0.05), followed by *E. coli* (13.0% *vs*. 11.6%), *Enterobacter* sp. (9.1% *vs*. 9.3%), *Serratia marcescens* (5.5% *vs*. 8.3%), *Proteus* sp. (4.3% *vs*. 7.4%), *Citrobacter koseri* (4.3% *vs*. 2.8%), *Hafnia alvei* (3.5% *vs*. 2.8%) and *Morganella morganii* (3.0% *vs*. 1.9%).

Among non-*Enterobacterales* GNB, *Pseudomonas* sp. were more frequently isolated from VAPs of COVID + (23.9% *vs*. 16.7%; p < 0.01) without difference in the proportion of respective species. Other bacteria identified were *Acinetobacter* sp. (3.2% *vs*. 1.9%), *Stenotrophomonas maltophilia* (3.5% vs. 3.7%), *Achromobacter* sp. (0.7% *vs*. 0.9%), *Haemophilus* sp. (4.3% *vs*. 8.3%; mainly *H. influenzae* 92.3% vs. 100% of *Haemophilus* sp.) and *Moraxella catarrhalis* (0.3% *vs*. 0.5%). *Burkholderia* sp., *Neisseria* sp. and *Prevotella* sp. were observed only in COVID +, in contrast to *Sphingomonas* sp. observed only in COVID-.

### Epidemiology of antibiotic resistance

Among GPB, the AST profile was characterized for all different species (*enterococci*, *Streptococci* including *S. pneumoniae*) without difference between the two clinical groups (Additional file 2) except for *Staphylococci* (Additional file 2: Table S1). For *Staphylococci*, no differences were observed for all the antibiotic families tested, including β-lactams, quinolones, glycopeptides, fosfomycin, fusidic acid, rifampicin, and cotrimoxazole except for aminoglycoside phenotype (0.7 *vs.* 7.4%; p < 0.05). Due to lack of power, none of these differences could be observed after stratification by bacterial species (coagulase-negative *Staphylococci, CNS, vs S. aureus*).

Among *Enterobacterales*, no differences between clinical groups were observed with respect to quinolone resistance (17.8% *vs.* 9.7%) (Table 3). A trend towards overrepresentation of fosfomycin resistance (29.8% *vs.* 15.0%) and overrepresentation of cotrimoxazole-resistant bacteria was observed in COVID + (18.5% *vs.* 6.1%; p < 0.05). This was also observed when stratified by species, for *K. pneumoniae* (39.6% *vs.* 0%; p < 0.05). Overrepresentation of aminoglycoside-resistant strains was observed in COVID- (13.9% *vs.* 20%; p < 0.01), due mainly to an association of tobramycin and amikacin resistance (3.5% *vs.* 11.4%; p < 0.01). Regarding beta-lactams, in COVID-, *E. coli* was more frequently resistant to amoxicillin and ticarcillin (8.7% *vs.* 28.6%; p < 0.05), *K. pneumoniae* was less resistant to cephalosporins (p < 0.05) and the *Enterobacter* cloacae complex overexpressed more frequently its cephalosporinase (27.4% *vs.* 60%; p < 0.05). Considering each *Enterobacterales*, no difference was observed with respect to quinolone or fosfomycin resistance (p > 0.05), whereas with respect to aminoglycoside-resistance, this difference in *Enterobacterales* was likewise observed in *S.marcescens* (55.1% *vs.* 87.5%; p < 0.05).

**Table 3:** AST profile per bacteria (*Enterobacterales*)

Enterobacterales	Whole-group			*Escherichia coli*			*Klebsiella pneumoniae/*sp.			*Enterobacter cloacae*/sp.			*Serratia marcescens*		
	COVID + (n; %)	COVID-(n; %)	p-value	COVID + (n; %)	COVID-(n; %)	p-value	COVID + (n; %)	COVID-(n; %)	p-value	COVID + (n; %)	COVID-(n; %)	p-value	COVID + (n; %)	COVID-(n; %)	p-value
β-lactam (n; % of evaluated in the respective group)	–*	–*	–*	–	–	< 0.05			< 0.05 < 0.05	–	–	–			–
WT	–*	–*	–*	27 (39.1)	6 (42.9)	–	29 (22.3)/49 (37.7)	9 (90.0)/12 (57.1)	< 0.05/–^a^	24 (47.1)/25 (46.3)	4 (40)/6 (50)	–/–	–		–
Cephalosporinase	–*	–*	–*	6 (8.7)	4 (28.6)	< 0.05	3 (2.3)/34 (26.2)	0 (0)/4 (19)	–/–	–	–	–	31 (93.9)	8 (88.9)	–
HC	–*	–*	–*	3 (4.3)	0 (0)	–	4 (3.1)/25 (19.2)	1 (10.0)/5 (23.8)	–/–	14 (27.4)/14 (25.9)	6 (60)/6 (50)	< 0.05/–	2 (6.1)	1 (11.1)	–
e-ESBL	–*	–*	–*	15 (21.7)	1 (7.1)	–	18 (13.8)/19 (14.6)	0 (0)/0 (0)	–/–^a^	9 (17.6)/9 (16.7)	0 (0)/0 (0)	–/–	–	–	–
HP	–*	–*	–*	18 (26.1)	3 (21.4)	–	–	–	–	–	–	–/–	–	–	–
CPE	–*	–*	–*	–	–	–	1 (0.8)/3 (2.3)	0 (0)/0 (0)	–/–	4 (7.8)/4 (7.4)	0 (0)/0 (0)	–/–	–	–	–
Aminoglycosides-resistant (n; % of evaluated in the respective group)	72 (13.9)	14 (20.0)	< 0.05	8 (11.6)	2 (14.3)	–	20 (0)/23 (17.6)	1 (10.0)/1 (4.8)	–/–	10 (20.8)/10 (19.6)	1 (11.1)/1 (9.0)	–/–	13 (44.8)	1 (12.5)	< 0.05
Quinolone-resistant (n; % of evaluated in the respective group)	60 (17.8)	7 (9.7)	–	18 (26.9)	2 (14.2)	–	18 (32.7)/22 (16.8)	1 (10)/1 (4.8)	–/–	12 (23.5)/12 (22.6)	3 (30)/3 (27.3)	–/–	0 (0)	0 (0)	–
Fosfomycin-resistant (n; % of evaluated in the respective group)	59 (29.8)	6 (15.0)	–^a^	2 (5.7)	0 (0)	–	17 (48.6)/40 (47.1)	2 (40)/3 (25)	–/–	6 (22.2)/6 (20)	1 (14.2)/1 (12.5)	–/–	–	–	–
Cotrimoxazole-resistant (n; % of evaluated in the respective group)	67 (18.5)	4 (6.1)	< 0.05	19 (28.4)	0 (0)	–	21 (39.6)/23 (18.4)	0 (0)/0 (0)	< 0.05/–	13 (26.0)/13 (24.5)	2 (20)/2 (18.2)	–/–	0 (0)	0 (0)	–

*WT* wild-type, *HC* hyper-expressed cephalosporinase, *e-ESBL* Extended-spectrum beta-lactamase producing enterobacterales, Hyper-expressed penicillinase, *CPE* Carbapenemase-producing enterobacterales, *: non evaluated

^a^Statistical tendency (p < 0.10)

Among the non-*Enterobacterales* GNB (Table 4), no difference between groups was observed regarding the antibiotic resistance profile of *H. influenzae*. Strains of *P. aeruginosa* also showed different phenotypes according to the clinical group: for beta-lactams, higher resistance to carbapenem (0.8% *vs.* 11.1%; p < 0.05); higher resistance to at least two aminoglycosides (1.4% *vs.* 17.7%, mainly gentamicin and tobramycin; p < 0.05), and higher resistance to fluoroquinolones in COVID- (7.1% *vs.* 53.6%; p < 0.05).

**Table 4:** AST profile per bacteria (non-*Enterobacterales* Gram-negative Bacilli)

A/*Haemophilus influenzae*/*Haemophilus* sp.		COVID + (n; %)	COVID- (n; %)	p-value
β-lactam (n = 31–33)	Penicillinase (n = 6–7)	6 (25.0)/7 (26.9)	0 (0)/0 (0)	–/–
	BLNAR (n = 3–3)	2 (8.3)/2 (7.7)	1 (33.3)/1 (33.3)	–/–
Quinolones (n = 30–32)	Resistant (n = 0–0)	0 (0)/0 (0)	0 (0)/0 (0)	–/–
Rifampicin (n = 17–17)	Resistant (n = 0–0)	0 (0)/0 (0)	0 (0)/0 (0)	–/–
Cotrimoxazole (n = 19–23)	Resistant (n = 11–12)	6 (26.1)/7 (28.0)	5 (62.5)/5 (62.5)	–^a^/–

B/*Pseudomonas aeruginosa*/*Pseudomonas* sp.		COVID + (n; %)	COVID- (n; %)	p-value
β-lactam (n = 161–165)	Carbapenem-resistant (n = 3–3)	1 (0.8)/1 (0.7)	2 (11.1)/2 (11.1)	< 0.05/ < 0.05
Aminoglycosides (n = 158–161)	Aminoglycosides-resistant (n = 12–12)	9 (6.4)/9 (6.4)	3 (17.7)/3 (17.7)	< 0.05/ < 0.05
Quinolones (n = 155–158)	Resistant (n = 25–25)	10 (7.1)/10 (7.0)	15 (53.6)/15 (53.6)	< 0.05/ < 0.05

C/*Stenotrophomonas maltophilia*		COVID + (n; %)	COVID- (n; %)	p-value
β-lactam (n = 25)	CAZ-Resistant (n = 18)	15 (71.4)	3 (75.0)	–
Quinolones (n = 42)	Resistant (n = 0)	0 (0)	0 (0)	–
Cotrimoxazole (n = 41)	Resistant (n = 0)	0 (0)	0 (0)	–

*BLNAR* Beta-lactamase-non-associated resistance, *CAZ* ceftazidime

^a^Statistical tendency (p < 0.10)

### Distribution of multiresistant bacteria by clinical group

Out of the bacteria analyzed, approximately one-fifth (18.4%) could be considered multidrug-resistant according to the previous definition (Table 5). COVID- patients were statistically more frequently infected with multidrug-resistant bacteria than COVID + (40.1% *vs.* 13.8%; p < 0.01). One third of these multiresistant GNB concerned enterobacteria (n = 55; 33.1%), CPE (7/55; 12.8%) or ESBL (48/55; 87.3%). For these multiresistant GNB, differential distribution could be observed between COVID + and COVID-, mainly associated with an over-representation in COVID + (12.2% *vs.* 2.8%; p < 0.05). Without difference between clinical groups, MDRs were due to ceftazidime resistant *Pseudomonadaceae*, CRAB and MRSA*.* Of note, in the present cohort, no GRE could be observed at diagnosis.

**Table 5:** Identified multi-resistant bacteria in diagnosis sample

		COVID + (n; %)	COVID- (n; %)	p-value
Multi-Resistant Bacteria (n; % of bacteria with complete antibiogram)	Presence (n = 166)	87 (13.8)	79 (40.1)	< 0.05
Methicillin-resistant *Staphylococcus aureus* (n; % of bacteria with complete antibiogram)	Presence (n = 16)	13 (10.0)	3 (12.0)	–
Ceftazidime-resistant *Pseudomonadaceae* (n; % of bacteria with complete antibiogram)	Presence (n = 21)	17 (12.0)	4 (22.2)	–
Beta-lactam-resistant *Acinetobacter* sp. (n; % of bacteria with complete antibiogram)	Presence (n = 5)	4 (33.3)	1 (50.0)	–
Vancomycin-resistant *Enterococcae* (n; % of bacteria with complete antibiogram)	Presence (n = 0)	0 (0)	0 (0)	–
Extended-spectrum beta-lactamase-producing *Enterobacterales* (n; % of bacteria with complete antibiogram)	Presence (n = 48)	46 (12.2)	2 (2.8)	< 0.05
Carbapenemase-producing *Enterobacterales* (n; % of bacteria with complete antibiogram)	Presence (n = 7)	7 (0.2)	0 (0)	–

## Discussion

This study is one of the major French studies of data from the first French wave of the COVID-19 pandemic. Unlike most publications focusing on this period in France, this study is multicentric, summarizing data from throughout France, including fifty-six hospitals. The results allow us to understand the bacterial presentation of VAP in a totally naïve human population during the first contact of the French population with SARS-CoV-2.

Influenza-associated VAPs are primarily caused by *S. pneumoniae, S. aureus,* and *H. influenzae*, whereas SARS-Cov2 demonstrated an association with *S. aureus* followed by *P. aeruginosa* and *Klebsiella* sp. [17]. Our cohort confirmed that VAPs associated with COVID had a different bacterial presentation and etiology than VAPs of other origins [17]. This could have a double interest. First, these bacteria are frequently associated with multidrug resistance/high resistance, which may require careful management of infection control in order to avoid disseminating multidrug-resistant bacteria (plasmid support, especially in CPE and E-ESBL, as in the current cohort) [18]. Second, preventive treatment of VAP, as suggested by national and international guidelines, may be more complicated in COVID + patients. Indeed, these patients more often require combined and/or longer treatment. For example, influenza superinfections are more frequently (compared to COVID +) associated with bacteria that could be controlled by more manageable antibiotic therapy (beta-lactam monotherapy) [19]. It is to note that the present cohort presented with a larger number of micro-organisms frequently implicated in community-acquired pneumonias (such as *Haemophilus* sp. and *Streptococccus pneumoniae*) more than in hospital-acquired pneumonia. This point could be considered as different from other descriptions in the literature but remains coherent, with the main species (*Staphyloccocus aureus f*or Gram-positive bacteria and *Pseudomonas aeruginosa* for Gram-negative bacteria) remaining found to be the most prevalent [20].

In a context of tension in health care institutions and hospitals, especially in intensive care units, it is important to analyze the proportion and spread of MDR bacteria. The present study has shown that a significant proportion (about one fifth) of MDR bacteria were observed during this period. Although this proportion is higher than usual in France, it is different from that observed by Moretti *et* *al.* in the Belgian cohort with the same proportions, but with regard to susceptible bacteria [19]. Despite these differences from one nation to another, it is crucial to observe that the presence of these MDR bacteria was higher in COVID-, which could have been due not only to the reinforcement and involvement of infection control specialists during this period, but also to the differences between this population (younger, less exposed to antibiotics, …) and the usual ICU population (both types of patients were recruited during the same period). This could also be explained by the multiple antibiotic therapies applied in patients with chronic respiratory diseases, which select resistant bacteria in all anatomical niches and in the airways, causing self-infection during mechanical ventilation [21]. Finally, it should be noted that even though it occurred 8 days after intubation, similarly to COVID- VAP in the literature, the diagnosis of VAP was earlier in COVID + patients than in COVID- patients, which could be another factor leading to a change in antibiotic susceptibility testing [22, 23]. This study also highlights the fact that some bacteria were mistakenly considered virulent enough to be treated. (*Lactobacillus*, *Alloscavordia*, alpha-hemolytic *Streptococci*, …), which may be associated with the globally high prescription of inappropriate antibiotic treatments during the early months of COVID pandemics [24]. In order to obtain an overview of the bacterial epidemiology of VAP during this very particular infectious period, all bacteria were considered to be involved in VAP (or as a therapeutic target) regardless of their involvement in the disease if they were alone. This decision could be justified by the fact that the clinicians could have modified their antibiotic therapy management to include this AST.

The study has limitations. First, the first French lockdown was a very particular time in 2020. At that time, clinical departments and medical analysis laboratories were faced with a heavy workload, resulting in logistical difficulties. To overcome these difficulties, while this study was conducted over a 1 month period, and considered cross-sectional at the time, some of the data were collected retrospectively during the summer and early fall of 2020. Even if the situation has evolved, this study benefits from the homogeneity of this first wave, during which only the original viral strain circulated in France, thereby limiting the possible impact of variants of concern (such as delta or omicron) [25]. Second, while this study includes many hospitals (academic and general), it cannot be considered comprehensive, insofar as the two largest teaching hospitals did not respond to the request for information. Nevertheless, this bias can be considered negligible, as the geographic distribution of the responding hospitals and their diversity provided a very good representation of the situation (Additional file 1: Fig. S1). Third, even if this study did not provide answers on the clinical and biological management of these patients during this period, the epidemiology described here could be considered robust because of its representativeness in the absence of ongoing antibiotic therapy at the time of sampling. This point is of great interest insofar as the development of antibiotic resistance during the clinical management of patients may have been considerably favored at that time, during which more than three quarters of patients worldwide received antibiotics, even in the absence of proven/suspected bacterial co-susceptibility (only 1 to 8%) [26].

## Conclusion

In conclusion, this study provided data on bacterial epidemiology and antibiotic resistance during the first wave of COVID pandemics. These observation calls for further studies focusing on the specific impact of these practice changes and assessing the impact of global pandemic management (including optimization of COVID management, vaccination, and development of viral variants).

## Supplementary Information

**Additional file 1: Figure S1.** Geographical origin of the data analysed in this study.

**Additional file 2: Table S1.** AST profile per bacteria.

## Data Availability

Supporting data is available upon request from the corresponding author.
