# Supplementary figures and images for: French national epidemiology of bacterial superinfections in ventilator-associated pneumonia in patients infected with COVID-19: the COVAP study
Source: Ann Clin Microbiol Antimicrob. 2023 Jun 28;22:50. doi: 10.1186/s12941-023-00603-0 (PMC10303851; doi:10.1186/s12941-023-00603-0)

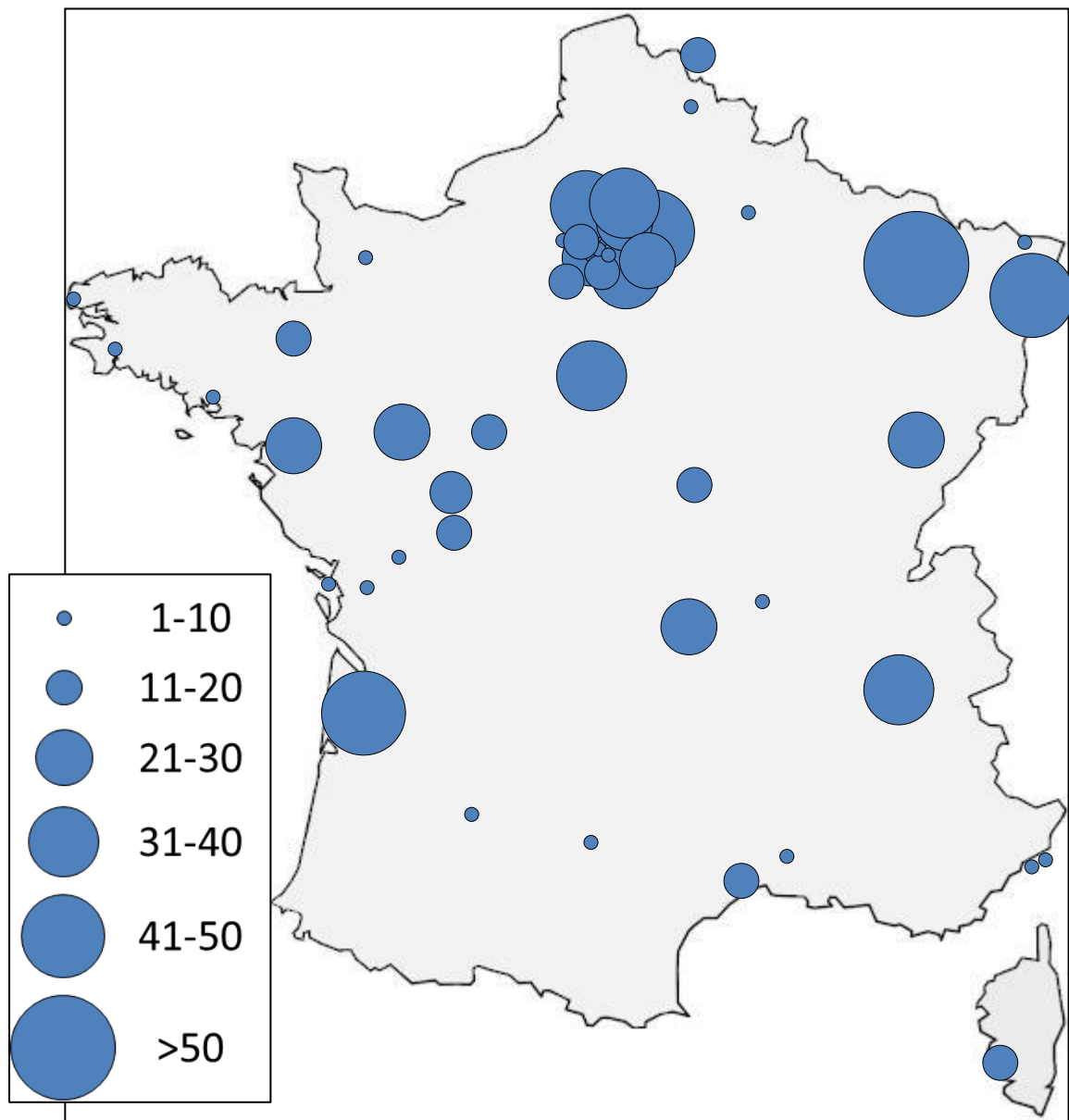

Supplement: Supplementary file 1 — Additional file 1: Figure S1. Geographical origin of the data analysed in this study. [file 12941_2023_603_MOESM1_ESM.pdf]
