# Supplementary material for: French national epidemiology of bacterial superinfections in ventilator-associated pneumonia in patients infected with COVID-19: the COVAP study
Source: Ann Clin Microbiol Antimicrob. 2023 Jun 28;22:50. doi: 10.1186/s12941-023-00603-0 (PMC10303851; doi:10.1186/s12941-023-00603-0)
Supplement: Supplementary file 2 — Additional file 2: Table S1. AST profile per bacteria. [file 12941_2023_603_MOESM2_ESM.docx]

**Table S1. AST profile per bacteria (*Staphylococcaeae*).**

|  | | **COVID+ (n; %)** | **COVID- (n; %)** | **p-value** |
| --- | --- | --- | --- | --- |
| β-lactam (n=129) | Resistance to methicillin | 19 (13.8) | 5 (22.7) | -^a^ |
| Aminoglycosides (n=165) | Resistance | 15 (10.8) | 3 (11.1) | - |
| Quinolones (n=158) | Resistance | 15 (11.2) | 4 (16.6) | - |
| Glycopeptides (n=64) | Resistance | 0 (0) | 0 (0) | - |
| Fosfomycine (n=139) | Resistance | 0 (0) | 0 (0) | - |
| Fusidic acid (n=142) | Resistance | 6 (5.0) | 2 (8.7) | - |
| Rifampicin (n=154) | Resistance | 1 (0.8) | 1 (4.4) | - |
| Cotrimoxazole (n=161) | Resistance | 10 (7.4) | 0 (0) | - |
| ^a^ Statistical tendency (p<.1) | | | | |
